# Interchangeability between factor analysis, logistic IRT, and normal ogive IRT

**DOI:** 10.3389/fpsyg.2023.1267219

**Published:** 2023-09-25

**Authors:** Eunseong Cho

**Affiliations:** Department of Business Administration, Kwangwoon University, Seoul, Republic of Korea

**Keywords:** item response theory, logistic model, normal ogive model, Monte Carlo simulation, normal distribution, logistic distribution, factor analysis

## Abstract

In existing studies, it has been argued that factor analysis (FA) is equivalent to item response theory (IRT) and that IRT models that use different functions (i.e., logistic and normal ogive) are also interchangeable. However, these arguments have weak links. The proof of equivalence between FA and normal ogive IRT assumes a normal distribution. The interchangeability between the logistic and normal ogive IRT models depends on a scaling constant, but few scholars have examined whether the usual values of 1.7 or 1.702 maximize interchangeability. This study addresses these issues through Monte Carlo simulations. First, the FA model produces almost identical results to those of the normal ogive model even under severe nonnormality. Second, no single scaling constant maximizes the interchangeability between logistic and normal ogive models. Instead, users should choose different scaling constants depending on their purpose in using a model and the number of response categories (i.e., dichotomous or polytomous). Third, the interchangeability between logistic and normal ogive models is determined by several conditions. The interchangeability is high if the data are dichotomous or if the latent variables follow a symmetric distribution, and vice versa. In summary, the interchangeability between FA and normal ogive models is greater than expected, but that between logistic and normal ogive models is not.

## Introduction

When dealing with discrete data (e.g., yes/no or Likert scale data), we can use either factor analysis (FA) or item response theory (IRT). Typical FA is for continuous data, but we can use FA for discrete data by assuming underlying continuous variables. There are various IRT models, most of which use logistic or normal ogive functions. That is, we have three models: FA, logistic IRT, and normal ogive IRT. Assume that we use them for two purposes: estimating parameters from given data and generating data from given parameters. Interchangeability is the degree to which models produce approximate results for each purpose when their apparent differences are removed. For example, if a transformation exists that makes the parameters estimated by one model identical to those estimated by another model from the same data, the two models are fully interchangeable for parameter estimation. If no statistical analysis is better than a random guess at distinguishing between the data generated by two models, the two models are fully interchangeable for data generation. We know that these three models are roughly interchangeable in some cases (Wirth and Edwards, 2007), but we know little about under what conditions and to what extent these models are interchangeable.

FA and IRT have different traditions and seemingly unrelated formulas. Nevertheless, scholars have begun to explain the relationship between FA and IRT (Lord and Novick, 1968; Bartholomew, 1983; Muthén, 1983). An explicit explanation was provided by Takane and de Leeuw (1987), who algebraically proved the equivalence of some FA models with some IRT models and presented formulas for transforming an FA model to an IRT model. These findings led Takane and de Leeuw (1987) to claim “[i]t is clear … that IRT and FA are two alternative formulations of a same model” (pp. 396–397). Modern scholars explain FA and IRT models within the same framework (Wirth and Edwards, 2007). However, few scholars have expressed a cautious view, suggesting that the claims of model equivalence are overgeneralized to areas outside what is substantiated by proof. The algebraic approach found in existing studies requires an assumption to obtain a solution. However, real-world data rarely satisfy this assumption. This study, through an empirical approach (i.e., Monte Carlo simulations), examines 3 weak links in the claim that FA and IRT models are interchangeable.

First, Takane and de Leeuw’s proof assumes that latent variables (i.e., factors in FA or abilities in IRT) follow a normal distribution. There is not yet a proof that assumes another distribution or a general proof that does not assume any distribution. This study empirically examines whether Takane and de Leeuw’s proof is robust to moderate or severe normality violations.

Second, Takane and de Leeuw’s proof applies to the normal ogive model instead of the logistic model. The normal ogive function is mathematically unwieldy because it does not have a closed form. Users typically use logistic models, which have simple formulas and produce approximate results to normal ogive models. What makes the parameters of the two models interchangeable is a scaling constant, often denoted as D . The values that are almost always used are 1.7 and 1.702 (Haley, 1952; Birnbaum, 1968). These values have been used unchallenged for decades, despite the lack of empirical evidence that it is the best choice for its original purpose. This study examines which value to use to maximize the interchangeability between models.

Third, few scholars have examined whether interchangeability for one purpose guarantees interchangeability for another. When existing studies have stated that one model is equivalent to or exchangeable with another model, they have rarely clarified the purpose of the models. For example, if two models are interchangeable when used to generate data, can we be sure that the two models are also interchangeable when used to estimate parameters? Few scholars have examined interchangeability from various angles.

This study addresses these issues through Monte Carlo simulations. Before explaining the simulations, the basic concepts are explained for readers unfamiliar with the topic.

## Literature review

### Probability distributions

#### Skewness and kurtosis

**Figure 1:**
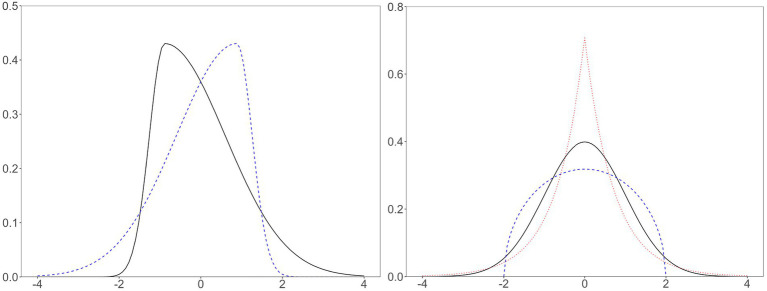
Skewed, normal, platykurtic, and leptokurtic distributions. Left solid line, positively skewed, skewness = 2; left dashed line, negatively skewed, skewness = −2; right solid line, normal distribution, kurtosis = 0; right dashed line, platykurtic distribution (i.e., Wigner semicircle distribution), kurtosis = −1; right dotted line, leptokurtic distribution (i.e., double exponential or Laplace distribution), kurtosis = 3. All distributions have zero mean and unit variance. The distributions on the left have zero kurtosis, and the distributions on the right have zero skewness.

When describing a probability distribution, we often use skewness and kurtosis (Figure 1) in addition to mean (i.e., μ ) and variance (i.e., $\sigma^{2}$ ). Skewness is defined as $E\left({\left(X-\mu \right)}^{3}\right)/\sigma^{3}$ , where E(.) is the expected-value operator. Kurtosis is defined as $E\left({\left(X-\mu \right)}^{4}\right)/\sigma^{4}-3$ . A distribution with negative kurtosis is platykurtic, and a distribution with positive kurtosis is leptokurtic.

#### Normal and logistic distributions

**Figure 2:**
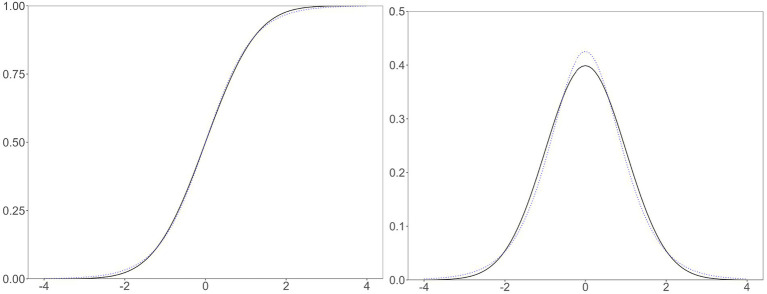
A comparison of normal and logistic distributions. Left, the cumulative density (i.e., ogive) functions; right, probability density functions; solid, the standard normal distribution; dotted, the logistic distribution with zero mean and scale constant of 1.702.

Of particular interest to this study are the normal and logistic distributions (Figure 2). Let Φ denote the standard normal ogive function: $\Phi \left(X\right)=\frac{1}{\sqrt{2\pi }}\int_{-\infty }^{X}\exp\left(-t^{2}/2\right)dt$ . Here, the ogive function is a cumulative density function. Let Ψ denote the standard logistic ogive function: Ψ(X)=1/(1+exp(−X))=exp(X)/(1+exp(X)) . In this case, the word ogive is usually omitted because typical users employ the logistic function without associating it with a logistic distribution (Savalei, 2006). Both distributions have zero skewness, but the logistic distribution has greater kurtosis (i.e., 1.2) than that of the normal distribution (i.e., zero).

**Figure 3:**
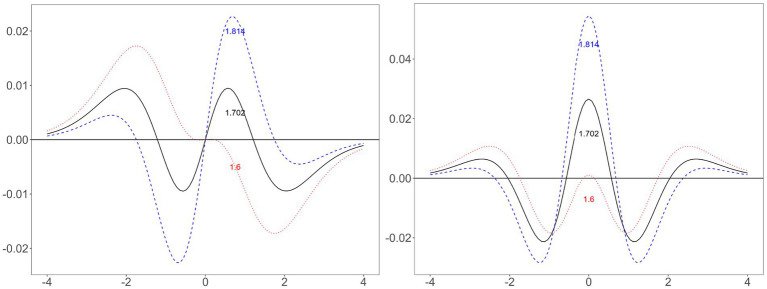
Difference between normal and logistic distributions when using three constants. Left, difference between logistic and normal ogive (i.e., cumulative) functions; right, difference between logistic and normal probability density functions; horizontal line containing zero: the standard normal distribution; solid line, logistic distribution using the scale constant of 1.702; dashed line, logistic distribution using the scale constant of 1.814; dotted line, logistic distribution using the scale constant of 1.6.

Statisticians have described distributions with different kurtosis values in several ways, and the normal and logistic distributions fit these descriptions. The logistic function has a thicker center (i.e., X near zero) and tails (i.e., beyond and around ±2) and less thick shoulders (i.e., around ±1) than those of the normal ogive function (Balanda and MacGillivray, 1988). The logistic distribution has a greater propensity to produce outliers than does the normal distribution (Westfall, 2014). The probability density function (PDF) of a normal distribution crosses that of a logistic distribution with the same mean and variance four times (Dyson, 1943). Since a PDF is the derivative of an ogive function, applying Dyson’s description to the ogive function, the logistic function minus the normal ogive function has four local minima or maxima with the signs +, −, +, and – (Figure 3). The different kurtosis values make the two functions different.

To quantify the difference, the logistic function has a maximum absolute error of approximately 0.01 from the normal ogive function (Haley, 1952); IRT studies describe the value of 0.01 as sufficiently small. For example, citing this value, Birnbaum (1968) argued that “any graph of a [normal ogive function] would serve equally well to illustrate [a logistic function]” (p. 399). Few studies explain this value using a numerical example, allowing the possibility that their readers misconceive it as a relative error of 1% rather than an absolute error. One of the X values at which the maximum absolute difference between the two functions is located is −2.044 if we use Haley’s (1952) scale constant: Φ(−2.044)≈0 .020 and Ψ(−1.702×2.044)≈0 .030, a relative difference of approximately 50%. Inputting an X value less than −2.044 decreases the absolute difference but increases the relative difference. For example, Φ(−3)≈0 .001 and Ψ(−1.702×3)≈0 .006. Whether this difference is small is debatable.

Let us review another reference point for determining whether 0.01 is a negligible size. Statisticians have devised dozens of closed-form functions that approximate the normal ogive function, most of which approximate the normal ogive function better than the logistic function does. For example, some have a maximum absolute error of approximately 0.0000001 (Shore, 2005). Unfortunately, these functions are more complex than the logistic function. The better they approximate the normal distribution, the more terms they have (Dombi and Jónás, 2018). Their complexity makes it difficult for humans to remember and understand them, although computers may have no problem calculating them. In summary, the logistic distribution has the advantage of simplicity, but it is not the best approximation of the normal distribution. This property can prevent IRT models that use the two functions from being fully interchangeable.

### FA and IRT models

#### FA models

This study uses a unidimensional FA model. Let us suppose there are n persons and k items. The continuous score of person $i\;\left(=1,\cdots ,n\right)$ for item j $\left(=1,\cdots ,k\right)$ (i.e., $X_{ij}^{*}$ ) has zero mean and unit variance. $X_{ij}^{*}$ is a linear combination of person i ’s latent variable (i.e., $\theta_{i}$ ) and an error (i.e., $e_{i}$ ): $X_{ij}^{*}=\lambda_{j}\theta_{i}+e_{i}$ , where $\lambda_{j}\left(\leq 1\right)$ is the factor loading. $\theta_{i}$ is drawn independently from a normal or nonnormal distribution with zero mean and unit variance. $e_{i}$ is independently drawn from a normal distribution with zero mean and variance $1-\lambda_{j}^{2}$ . We can transform the continuous score $X_{ij}^{*}$ into a discrete score $X_{ij}$ by using thresholds. If there are two categories, $X_{ij}$ is one of $\left\{0,1\right\}$ . Let $\tau_{j}$ denote the threshold of item j . We obtain $X_{ij}=0$ if $X_{ij}^{*}<\tau_{j}$ and $X_{ij}=1$ otherwise. If there are C categories, $X_{ij}$ is one of {0,1,⋯,C−1} . Let $\tau_{jc}$ denote the c th threshold of item j , where c is one of $\left\{0,1,\cdots ,C\right\}$ . The first and last thresholds are trivial: $\tau_{j0}=-\infty$ and $\tau_{jC}=\infty$ . We obtain $X_{ij}=c$ if $\tau_{jc}\leq X_{ij}^{*}<\tau_{j\left(c+1\right)}$ .

#### IRT models

##### Normal ogive

If there are two categories, the two-parameter model is $P\left(X_{ij}=1|\theta_{i}\right)=\Phi \left(\alpha_{j}\theta_{i}-\beta_{j}\right)$ , where $\alpha_{j}$ is the slope or discrimination parameter of item j and $\beta_{j}$ is the location or difficulty parameter of item j . We automatically obtain $P\left(X_{ij}=0|\theta_{i}\right)=1-P\left(X_{ij}=1|\theta_{i}\right)$ . If there are C categories, the two-parameter model is $P\left(X_{ij}\geq c|\theta_{i}\right)=\Phi \left(\alpha_{j}\theta_{i}-\beta_{jc}\right)$ , where $\beta_{jc}$ is the c th ( c=1,…,C−1 ) location parameter of item j . We obtain $P\left(X_{ij}=c|\theta_{i}\right)=P\left(X_{ij}\geq c|\theta_{i}\right)-P\left(X_{j}\geq c+1|\theta_{i}\right)$ , where $P\left(X_{j}\geq C|\theta_{i}\right)=0$ .

##### Logistic

Birnbaum’s (1968) two-parameter model describes dichotomous item scores as $P\left(X_{ij}=1|\theta_{i}\right)=\Psi \left(D\left(\alpha_{j}\theta_{i}-\beta_{j}\right)\right)$ , where D is a scaling constant. When C is greater than 2, the two-parameter model is $P\left(X_{ij}\geq c|\theta_{i}\right)=\Psi \left(D\left(\alpha_{j}\theta_{i}-\beta_{jc}\right)\right)$ .

### Interchangeability between models

#### Between logistic and normal ogive models

##### Scaling constant

The link connecting the logistic and normal ogive models is the scaling constant D . The purpose of this constant is to make the results of the two models as approximately equal as possible. The first to use this constant in logistic models was Birnbaum (1968), who used a value of 1.7, citing Haley (1952). Hailey’s original value was 1.702 (Camilli, 1994), the value used in this study. Three other values (i.e., 1.6, 1.749, and 1.814) have been proposed as scaling constants. Furthermore, further thought reveals that any value between 1.6 and 1.814 can be used as a scaling constant. This study is interested in which is the best but first describes the rationale each value is based on and how each approximates the two distributions.

##### Review of existing rationales

Let us start with the old constants. The easiest rationale for approximating the normal and logistic distributions is to equalize the variances of the two distributions, which gives 1.814 ( $\approx \pi /\sqrt{3}$ ). However, this value creates excessively large nontail errors (Figure 3). Haley’s (1952) idea was to minimize the maximum absolute error, which makes the maximum tail and nontail error have the same value (Camilli, 1994). Therefore, the value he obtained, 1.702, makes the difference between the two distributions oscillate between the maximum absolute errors (i.e., approximately −0.01 and.01). Amemiya (1981) calculated 13 values from normal and logistic distributions: 1.6 is the value he found by trial and error to minimize the difference between the two distributions. Of the 13 values Amemiya (1981) reviewed, 11 are in the nontail region (i.e., X = 0, 0.1, 0.2, …, 0.9, and 1), and only two are in the tail (i.e., X = 2 and 3). These constants are based on a relatively simple rationale.

Few scholars have attempted to find a better value than these older values. An exception is Savalei (2006), who suggested a value of 1.749 that minimizes the Kullback–Leibler (KL) information (Kullback and Leibler, 1951). Savalei did not directly demonstrate how minimizing the KL information is related to maximizing the interchangeability between the logistic and normal ogive models. In any case, her study is based on more sophisticated statistical rationales than those of existing values, and our best knowledge at this moment expects that 1.749 maximizes model interchangeability. An interesting point raised by Savalei is that the KL divergence when the normal ogive function is approximated by the logistic function differs from the KL divergence when the logistic function is approximated by the normal ogive function (the value that minimizes the latter is 1.814). This asymmetry suggests that the departure and arrival models should be clear (i.e., from normal ogive to logistic) when discussing interchangeability.

##### Issues illustrated by graphs

An intuitive understanding can originate from graphs (Figure 3). First, choosing a constant involves a trade-off. The difference between the two ogive functions has four local minima or maxima (i.e., X≈ –2, −0.5, 0.5, and 2). Let the errors near −2 and 2 be the tail errors and the errors near −0.5 and 0.5 be the nontail errors. Minimizing one of these comes at a sacrifice of the other. A large constant (e.g., 1.814) reduces the tail errors at the cost of increasing the nontail errors; a small constant (e.g., 1.6) does the opposite. The difference between these constants is how much weight to give to the tail and nontail errors.

The above discussion suggests that using 1.702 makes sense only in a special case, i.e., when we give equal weight to the tail and nontail errors. Birnbaum’s (1968) explanation that using 1.702 ensures that the maximum errors never exceed 0.01 (i.e., an easy number to remember) appeals to human intuition but misses the point. We should give these two errors optimal weights, not equal weights. Nontail regions are more frequent than tail regions in a bell-shaped distribution, so if we weight frequencies, the nontail errors outweigh the tail errors. However, the tail errors may exert a greater impact than the nontail errors do. It is difficult to predict which of these two scenarios is correct in the IRT model.

Second, minimizing differences in the ogive functions differs from minimizing differences in the PDFs. One might think that both questions have the same answer (Cook, 2010). However, a comparison of the left and right sides of Figure 3 reveals that a constant that performs well in one case may perform poorly in another. In the ogive function, 1.702 has smaller maximum errors than those obtained with 1.6 or 1.814. However, in the PDF, 1.702 has a larger maximum error than that of 1.6. However, that does not mean that 1.6 is the best. Little is known about which value minimizes the PDF difference between the normal and logistic distributions.

##### Rationales not studied by existing studies

Little research has been done to examine the difference between the normal and logistic distributions from various angles. Haley (1952) focused on minimizing the maximum absolute error for the ogive functions. First, the usual approach to error minimization is to minimize the sum of all errors, whether absolute or squared. Second, which scaling constant minimizes the difference in PDFs has not yet been examined. Study 1 addresses these issues.

In existing studies, logistic and normal distributions instead of logistic and normal ogive models have been the source of comparison. The two problems may have different answers. Probability distributions produce continuous outputs, whereas typical IRT models produce discrete outputs. Understanding logistic and normal distributions is only a means to understanding the difference between logistic and normal ogive models. In addition to an indirect examination through probability distributions, this study directly examines our ultimate concern, that is, the interchangeability between logistic and normal ogive models. Studies 2 through 4 address this issue.

#### Between the FA and normal ogive models

Takane and de Leeuw’s (1987) proof suggests that we can transform FA models into two-parameter normal ogive models, and vice versa, if the latent variables follow a normal distribution. The formulas for transforming the parameters of the FA model to those of the normal ogive model are as follows:

(1) $$\alpha_{j}=\lambda_{j}/\sqrt{1-\lambda_{j}^{2}}\;and\;\beta_{jc}=\tau_{jc}/\sqrt{1-\lambda_{j}^{2}}$$.

Rearranging these formulas creates the opposite formulas:

.(2) $$\lambda_{j}=\alpha_{j}/\sqrt{1+\alpha_{j}^{2}}\;and\;\tau_{jc}=\beta_{jc}/\sqrt{1+\alpha_{j}^{2}}$$

The formulas are proven only when the latent variables follow a normal distribution. Latent variables in the real world are not exactly normally distributed. If the formulas do not hold even for data that slightly violate normality, it is difficult to apply them to parameter estimation and data generation for practical purposes. On the other hand, if the formulas hold for data that severely violate normality, we should use them more aggressively than we do now.

### Approaches to model interchangeability

#### Isolating model interchangeability from others

We can use an FA or IRT model in two directions: to estimate parameters from given data and to generate data from given parameters. Most users use a model for parameter estimation, so it makes sense for academic research to focus on parameter estimation as well. However, research focusing on parameter estimation may have difficulty isolating whether differences in parameter estimates between models are due to differences in models or other causes. The FA, logistic, and normal ogive models have their own estimation techniques and, in many cases, dedicated software. For example, Wirth and Edwards (2007) used weighted least squares for categorical data (WLS) and modified WLS for categorical data (MWLS) for the FA model, the expectation–maximization (EM) technique for the logistic model, and the Markov chain Monte Carlo estimation technique for the normal ogive model. In this case, it is challenging to isolate whether the differences in the parameter estimates are due to different models or different estimation techniques. Their work also suggests that the effect of different estimation techniques overpowers that of different models. Parameter estimates of the FA model by MWLS were approximate to those of the logistic and normal ogive models but were meaningfully different from those of the FA model via WLS. In summary, the above discussion suggests that isolating model interchangeability from other causes requires using only one model for parameter estimation or omitting it.

#### Overview of this study

##### Each study

This study examines model interchangeability from various angles through six studies (Table 1). First, Study 1 examines only probability distributions instead of using IRT models, as previous studies did (Haley, 1952; Amemiya, 1981; Savalei, 2006). Second, Study 2 examine model interchangeability in parameter estimation and use a logistic model for parameter estimation. Third, Studies 3 and 4 examine model interchangeability in data generation and use all three models for data generation. Among these, additional explanation is needed for the Kolmogorov–Smirnov (KS) test to be performed in Study 3.

**Table 1:** Summary of each study.

	Data generation	Parameter estimation	Focus
Study 1	None	None	Probability distribution
Study 2	Normal ogive	Logistic	Scaling constant
Study 3	FA, logistic, normal ogive	None	Kolmogorov–Smirnov test
Study 4	FA, logistic, normal ogive	None	Graphical analysis

FA, factor analysis.

##### KS test

Study 3 performs the KS test, which tests whether two pieces of sample data are generated from the same probability distribution. This study proposes a weak criterion and a strong criterion for interchangeability by using the KS test. The weak criterion directly uses the KS test. First, one dataset is generated from each of the two models. Second, the KS test is performed on the two datasets. If there is no statistically significant difference, the two models pass the weak criterion for interchangeability. The original KS test targets continuous data and produces conservative results when applied to discrete data (Conover, 1972). To address this issue, R’s dgof package (Arnold and Emerson, 2011) provides *p* values calculated by using Monte Carlo simulations.

The strong criterion involves a blind test. For example, suppose that three sets of data are generated from two models, and two persons bet on which are generated from the same model. One makes random guesses, and the other uses statistical information (i.e., statistical guess). This person realizes that the KS statistic contains information even if there is no significant difference in the statistics of the three datasets. After comparing the KS statistics of the three datasets, this person guesses that the datasets with the smallest statistic were generated from the same model. If this statistical guess cannot outperform random guessing, the two models are considered fully interchangeable; otherwise, model interchangeability is measured by how much the statistical guess outperforms random guessing.

## Study 1: Finding scaling constants from probability distributions

Study 1 addresses which scaling constant minimizes the difference between logistic and normal distributions. Current knowledge on this issue is limited in two ways: First, it covers only the ogive functions of the two distributions, not their PDFs; second, the knowledge is derived by minimizing the maximum error (Haley, 1952), not the total error (i.e., the sum of absolute or squared errors), which is a typical concern in error minimization.

Haley’s choice may have been due to the computational advantage of the former over the latter. A human can find *D* that minimizes the maximum error (i.e., $\underset{D}{\min}\underset{\theta }{\max}|\Psi \left(D\theta \right)-\Phi \left(\theta \right)|$ ) without a computer. Differential calculus simplifies the calculation, and the known approximate value of 1.814 enables us to obtain a convergent solution in only three iterations (Camilli, 1994). However, minimizing the total error requires integration [e.g., $\underset{D}{\min}\int_{-\infty }^{\infty }{\left(\Psi \left(D\theta \right)-\Phi \left(\theta \right)\right)}^{2}d\theta$ ]; its formula is not in a closed form and is challenging for a human to compute without a computer. Haley’s (1952) solution of *D* = 1.702 may be due to his limited access to a computer.

This study takes a computational approach. First, the scaling constants that minimize the maximum absolute error of the two ogive functions and PDFs [i.e., $\underset{D}{\min}\underset{\theta }{\max}|\Psi \left(D\theta \right)-\phi \left(\theta \right)|$ ] are obtained, where ψ is the PDF of the logistic distribution and ϕ is the PDF of the standard normal distribution. Second, the scaling constants that minimize the sum of the absolute [i.e., $\int_{-\infty }^{\infty }|\Psi \left(D\theta \right)-\Phi \left(\theta \right)|d\theta$ and $\int_{-\infty }^{\infty }|\psi \left(D\theta \right)-\phi \left(\theta \right)|d\theta$ ] and squared (i.e., $\int_{-\infty }^{\infty }{\left(\Psi \left(D\theta \right)-\Phi \left(\theta \right)\right)}^{2}d\theta$ and $\int_{-\infty }^{\infty }{\left(\psi \left(D\theta \right)-\phi \left(\theta \right)\right)}^{2}d\theta$ ) errors are obtained.

### Methods

Obtaining the value that minimizes the maximum absolute error is a double optimization problem (i.e., $\underset{D}{\min}$ and $\underset{\theta }{\max}$ ). First, this study obtains the maximum absolute error (i.e., $\underset{\theta }{\max}$ ) by using R’s (R Core Team, 2023) optimize function. Second, the *D* value that minimizes (i.e., $\underset{D}{\min}$ ) these maximum absolute errors is obtained by a brute-force approach. This study obtains the maximum absolute errors of each using *D*’s in the 0.0001 interval between 1.6 and 1.8 and then reports the value that minimizes the maximum absolute errors.

Obtaining the value that minimizes the sum of the absolute or squared errors involves brute-force optimization. To obtain the integral, this study uses R’s integrate function, which relies on the Quadpack package (Piessens et al., 2011). All code used in this study is publicly.^1^

### Results and discussion

#### Results

Table 2 shows the results: 1.7017 represents Haley’s (1952) constant to four decimal places (Camilli, 1994).

**Table 2:** Constants that minimize the difference between the two distributions (Study 1).

	Maximum absolute error	Sum of errors	
		Absolute	Squared
Ogive function	1.7017	1.7062	1.7010
Probability density function	1.6034	1.6294	1.6267

#### Explanation of unexpected results

The values of the constants from the ogive functions are approximate, but the constants from the PDFs are not. The ogive function difference essentially has only two components, the tails and nontails, and minimizing the maximum error is equivalent to equalizing the maximum tail error and the maximum nontail error. The PDF difference has three components: the tails, shoulders, and center (Figure 3). Minimizing the maximum error simply involves minimizing the error in the area where the maximum error is located. According to further analysis, the maximum error is not in the tails for *D* values ranging between 1.6 and 1.8. If *D* is less than 1.677, the shoulders have the maximum error; otherwise, the center has the maximum error. Therefore, minimizing the maximum error between *D* = 1.6 and 1.677 minimizes the errors in the shoulders; the errors in the tails and center are irrelevant.

However, minimizing the sum of errors considers all areas. For example, minimizing the sum of the absolute errors involves minimizing the area between the curve and the line at *y* = 0 in the right graph of Figure 3. This minimization requires that each area be of a similar size. *D* = 1.6 creates an excessively small center area and excessively large tail areas; a constant greater than 1.6 increases the center area and decreases the tail areas.

#### Need for additional simulations

One may question the generalizability of Study 1. Study 1 considered only probability distributions and did not examine IRT models by using these distributions. Although the probability distributions are continuous, IRT models use them to generate or explain discrete item scores. In the process, a new factor that we are unaware of may intervene. Study 2 addresses this issue.

## Study 2: Finding scaling constants from IRT models

In existing studies (Haley, 1952; Amemiya, 1981; Savalei, 2006) and in Study 1, only normal and logistic distributions have been examined. This approach is indirect because IRT users are interested in models, and probability distributions are merely a means of describing models. There is no self-evident criterion as to which of these studies’ rationales, such as the maximum error, equal variances, or the KL information, are best applied to the interchangeability of IRT models. Study 2 addresses this issue by directly examining normal ogive and logistic models.

### Methods

The simulation has 2 conditions. Namely:

- Number of categories (*C*): dichotomous = 2 and polytomous = 5.

Each condition has 100,000 iterations. For each iteration, unidimensional data with a sample size of 10,000 and 10 items are generated by using the two-parameter normal ogive model. To increase the reality of the simulation, this study randomizes parameter values by drawing them independently for every item in every iteration. The discrimination parameter of each item (i.e., $\alpha_{j}$ ) is drawn from a uniform distribution between 0.6 and 1.4. These values correspond to factor loadings (i.e., $\lambda_{j}$ ) of 0.514 and 0.814 (Equation 2), respectively. These values are approximately 0.5 and 0.8, respectively, which Yang and Green (2015) claim to be typical factor loadings in FA.

In the dichotomous condition, the location parameter of each item (i.e., $\beta_{j}$ ) is drawn from a uniform distribution between-1.3 and 1.3. Using these values makes the expected proportion of each response category (i.e., 0 and 1) range between 0.179 and 0.821 in the normal distribution. In the polytomous condition, this study creates four parameters of each item (i.e., $\beta_{j1}$ , $\beta_{j2}$ , $\beta_{j3}$ and $\beta_{j4}$ ) in two steps. First, four values are drawn from a uniform distribution between-2 and 2 and then sorted in ascending order. Second, this study subtracts 0.2 from the first, subtracts 0.1 from the second, adds 0.1 to the third, and adds 0.2 to the fourth. These values make the average proportion of each category (i.e., 0, 1, 2, 3, and 4) 0.184, 0.193, 0.245, 0.193, and 0.184, respectively, in the normal distribution. However, these numbers are only averages, and the proportions of each dataset vary.

The latent variable of each person (i.e., $\theta_{i}$ ) is independently drawn from a standard normal distribution. The $\theta_{i}$ values are then substituted into the normal ogive model using the preceding parameters to generate data. The data are used to estimate the parameters of the logistic model by using the EM technique and the mirt package (Chalmers, 2012). Then, in a similar method to that used in Study 1, the value that minimizes the difference between the parameter used to generate the data and the estimated parameter is determined.

### Results and discussion

#### Results

Table 3 shows the results.

**Table 3:** Optimal scaling constant when the data generated by a normal ogive model are estimated by a logistic model (Study 2).

	Absolute error	Squared error
Dichotomous (*C*= 2)	1.7343	1.7387
Polytomous (*C*= 5)	1.7462	1.7525

*C*: the number of categories.

#### Explanation of unexpected results

##### Values greater than 1.702

A reasonable expectation is that the nontails are more important in parameter estimation than are the tails because the nontails are more frequent than are the tails. Study 1 also suggested that the scaling constant that minimizes the PDF difference between the normal and logistic distributions (i.e., 1.627 and 1.629) is less than 1.702. However, Study 2 produced results in the opposite direction. The scaling constants that make the parameter estimates of the logistic model most approximate to those of the normal ogive model are greater than 1.702: the values are 1.739 in the dichotomous condition and 1.753 in the polytomous condition according to the squared error criterion. A possible explanation is that the tails have a sufficiently large impact on the parameter estimates to offset their low frequency. The tails contain outliers, “which usually exert disproportionate influence” (Aguinis et al., 2013, p. 2) on parameter estimates. For example, adding an outlier to randomly generated two-variable data can turn a near-zero correlation into a near-perfect correlation (Trauth, 2017). As reviewed earlier (Figure 3), a large scaling constant reduces the tail errors rather than the nontail errors.

##### Number of categories

IRT users have been using the same scaling constant regardless of the number of categories of items (i.e., dichotomous or polytomous). However, the results suggest that the scaling constant that produces the most approximate parameter estimates to the normal ogive model differs between dichotomous and polytomous data. A possible explanation is that categories of dichotomous data do not specialize in either the tails or nontails, but those of polytomous data do. For example, in data with response categories of 0 and 1, both categories have one tail and share the nontail. Therefore, low weights for the tails do not severely increase the estimation error for 0 and 1. However, in data with response categories of 0, 1, 2, 3, and 4, only categories at both ends (i.e., 0 and 4) have tails, and the rest (i.e., 1, 2, and 3) focus on the nontails. Therefore, low weights for the tails can increase the estimation error for 0 and 4.

#### Need for additional simulations

Studies 1 and 2 focused only on the scaling constant. Study 3 compares the three models, including the FA model.

## Study 3: Model interchangeability in terms of the KS test

Study 3 answers three questions. First, it examines whether Takane and de Leeuw’s (1987) proof that the FA model is equivalent to the normal ogive model holds even if the data moderately or severely violate normality. Second, Study 3 examines which scaling constants make the logistic model produce results that best approximate those of the normal ogive model. Third, Study 3 examines how well the results of the logistic model approximate those of the FA and normal ogive models when using the best-performing scaling constant.

### Methods

#### Simulation design and data generation

The simulation has a 2 * 5 full factorial design with 10 conditions. Namely:

- Number of categories (*C*): dichotomous = 2 and polytomous = 5, and
- Distribution of latent variables: normal, skewed, platykurtic, leptokurtic and severe.

Each of the five distributions has the following skewness (front) and kurtosis (back): normal (0, 0), skewed (2, 0), platykurtic (0, −1.2), leptokurtic (0, 7), and severe (3, 21). Existing studies (Lee et al., 2020) used values of (2, 7) for moderate normality violation and values of (3, 21) for extreme violation. This study divides the first values into values for the skewed and leptokurtic conditions and uses the second values as they are. Moreover, the platykurtic condition is added by using the values of the uniform distribution. However, this distribution differs from the uniform distribution because this study creates nonnormal distributions by transforming the normal distribution (Fleishman, 1978).

The sample size (i.e., *n*) is 10,000. This study focuses on large samples because preliminary research suggests that small samples produce irregular results, probably due to sampling errors. This study performs 20,000 iterations for each condition. Each iteration uses the parameter values described in Study 2. Each person’s latent variable is independently drawn from a distribution with zero mean and unit variance and a predetermined skew and kurtosis. Each model produces two item scores. Item scores generated from the same model are identical except for randomness. For example, two item scores from the FA model are generated by using the same factor loading, thresholds, and latent values; the only difference is the errors.

#### Determining interchangeability

Only the first of the two item scores of each model is used for comparison between models. The KS statistic and *p* value are recorded for each of the 13 models (i.e., 1 FA, 1 normal ogive, and 11 logistic). If the p value between any models is less than 0.05, those models fail to satisfy the weak criterion for interchangeability.

An index, denoted as *b*, quantifies how much better a statistical guess is than a random guess in a blind test. *b* ranges between −1 (i.e., always incorrect) and 1 (i.e., always correct), where 0 is the expected value of random guessing. The blind test consists of two comparisons. For example, suppose we compare Model A and Model B. Each model has two parallel item scores: Model A has A1 and A2, and Model B has B1 and B2. The first comparison focuses on A1. The KS statistic between A1 and A2 is subtracted from the KS statistic between A1 and B1. If the subtracted value is negative, the statistical guess is correct, and *b* is 0.5. If the value is positive, the statistical guess is incorrect, and *b* is −0.5. If the value is zero, no statistical guess is made, and *b* is 0. The second comparison focuses on B1. The KS statistic between B1 and B2 is subtracted from the KS statistic between B1 and A1. If the subtracted value is negative, *b* is 0.5; if it is zero, *b* is 0; and if it is positive, *b* is −0.5.

### Results and discussion

#### Results

There was no case showing a statistically significant difference in the KS test. The KS statistics between scores generated by using the same model (i.e., parallel scores) were 0.0054 or 0.0055 on average. The KS statistic between the FA and normal ogive models was 0.0055. The KS statistic between the FA and logistic models ranged from 0.0062 to 0.0073, depending on the scaling constant used, and the KS statistic between the normal ogive and logistic models also ranged from 0.0062 to 0.0073. In summary, all models pass the weak criterion for exchangeability.

Table 4 shows the blind test results, i.e., the strong criterion of interchangeability. The comparison between the FA and logistic models is omitted except for the case of *D* = 1.70 because it has a pattern similar to that between the normal ogive model and the logistic model. Even in the latter comparison, the results for *D* = 1.65 and 1.66 are omitted to save space. First, the results of the FA and normal ogive models have near-zero values, suggesting that the data generated by the two models are indistinguishable. Second, the results of the normal ogive and logistic models vary depending on the conditions and scaling constants used. This study considers 0.05 as the criterion for passing the blind test. In dichotomous data, the logistic models using the optimal scaling constant under the normal, platykurtic, and leptokurtic conditions meet this criterion, but none of the logistic models meet the criterion under the skewed and severe conditions. In polytomous data, none of the logistic models meet this criterion.

**Table 4:** Statistical guessing vs. random guessing (Study 3).

	FA-NO	FA-LO^*^	NO-LO using different scaling constants								
			1.67	1.68	1.69	1.70	1.71	1.72	1.73	1.74	1.75
Dichotomous (number of categories = 2)											
Normal	−0.007	0.017	0.056	0.036	0.022	0.015	0.002	0.021	0.027	0.046	0.065
Skewed	0.000	0.085	0.113	0.107	0.085	0.081	0.080	0.075	0.096	0.094	0.096
Platykurtic	−0.001	0.043	0.102	0.080	0.060	0.034	0.035	0.035	0.037	0.023	0.035
Leptokurtic	0.008	0.030	0.047	0.027	0.023	0.030	0.049	0.080	0.105	0.146	0.182
Severe	−0.005	0.098	0.084	0.077	0.083	0.104	0.130	0.167	0.203	0.252	0.307
Polytomous (number of categories = 5)											
Normal	0.011	0.177	0.355	0.284	0.232	0.171	0.144	0.110	0.096	0.099	0.124
Skewed	−0.006	0.238	0.332	0.294	0.253	0.229	0.208	0.196	0.207	0.224	0.255
Platykurtic	0.004	0.160	0.331	0.274	0.208	0.158	0.116	0.075	0.065	0.062	0.075
Leptokurtic	0.003	0.289	0.435	0.379	0.327	0.283	0.264	0.255	0.264	0.282	0.299
Severe	−0.001	0.423	0.523	0.480	0.447	0.436	0.414	0.433	0.440	0.485	0.519

FA, the factor analysis model; NO, the normal ogive model; LO, the logistic model; ^*^, the logistic model using 1.7 as the scaling constant. The blind test result can have a value between −1 and 1, and the higher the value is, the greater the difference between the two data generation models. A near-zero value means that the data generated by the two models are indistinguishable. The underlined values are the scaling constants that perform the best among the different scaling constants in each condition.

#### Explanation of unexpected results

##### Equivalence of the FA and normal ogive models

A reasonable expectation is that the FA and normal ogive models will produce distinguishable data, at least for severe violations of normality. However, even under the severe violation condition, the blind test results produced negative values (i.e., −0.005 and − 0.001), indicating that the data generated by the two models cannot be distinguished via the KS statistic. This result provides empirical evidence that the two models are equivalent regardless of normality violations.

##### Asymmetric use of scaling constants

For decades, IRT users have applied the same constant for parameter estimation and data generation. Therefore, a reasonable expectation is that the scaling constant performed best in Study 2 and in Study 3. However, except for the dichotomous platykurtic condition, the models that produced the best results in the blind test were those that used a scaling constant (i.e., between 1.68 and 1.74) smaller than the optimal constant (i.e., 1.734 to 1.753 depending on the condition) in Study 2. A possible explanation is that the tails in data generation are less important than those in parameter estimation. Suppose two persons A and B each have $\theta_{A}=-2$ and $\theta_{B}=-4$ . Both A and B belong to the tail, but B is more extreme than A. In parameter estimation, B has a greater influence than A. However, in data generation, if the location parameter is not extreme, A and B generate the same data. Existing studies have emphasized the disproportionate influence of outliers on parameter estimation but have rarely mentioned their influence on data generation (Aguinis et al., 2013). Different mechanisms operate for parameter estimation and data generation.

#### Need for an additional simulation

Study 3 suggests that the distribution of latent variables affects the optimal scaling constant but rarely answers why. Study 3 analyzes only the output of data generation instead of its process, thereby providing no clue as to why different distributions (e.g., normal vs. skewed) perform differently. Study 4 addresses this issue.

## Study 4: How parameters affect the data generated by each model

Study 4 addresses the unanswered questions raised by Study 3. First, Study 4 compares data generated by the FA, normal ogive, and logistic models for given parameters. To simplify the comparison, Study 4 uses the data generated by the normal ogive model as reference data and assesses the difference between the data generated by other models and the reference data. Study 3 had randomized parameters, but Study 4 uses predetermined parameters. This design enables us to observe how changes in parameters affect the data generated by each model.

Second, Study 4 compares data generated by the logistic models using different scaling constants under various distributions. To simplify the comparison, Study 4 uses only the largest (i.e., 1.74), the smallest (i.e., 1.68), and the middle (i.e., 1.71) scaling constants that produced good performance in Study 3. Moreover, Study 4 focuses on dichotomous data to save space.

### Methods

The simulation has 5 * 2 * 5 = 50 conditions. Namely:

- Distribution of latent variables: normal, skewed, platykurtic, leptokurtic and severe;
- Discrimination parameter ( α ) values: 0.6 and 1.4;
- Location parameter ( β ) values: −1, −0.5, 0, 0.5, and 1.

Each condition is used to generate one million latent variables with zero mean, unit variance and predetermined skewness and kurtosis. These latent variables are inputted into the FA, normal ogive, and logistic models with scaling constants of 1.68, 1.71, and 1.74 to generate single-item dichotomous data. To simplify the comparison, the value obtained by subtracting the proportion of response category 1 generated by the normal ogive model from the proportion of response category 1 generated by the FA and logistic models is reported.

### Results and discussion

#### Results

**Figure 4:**
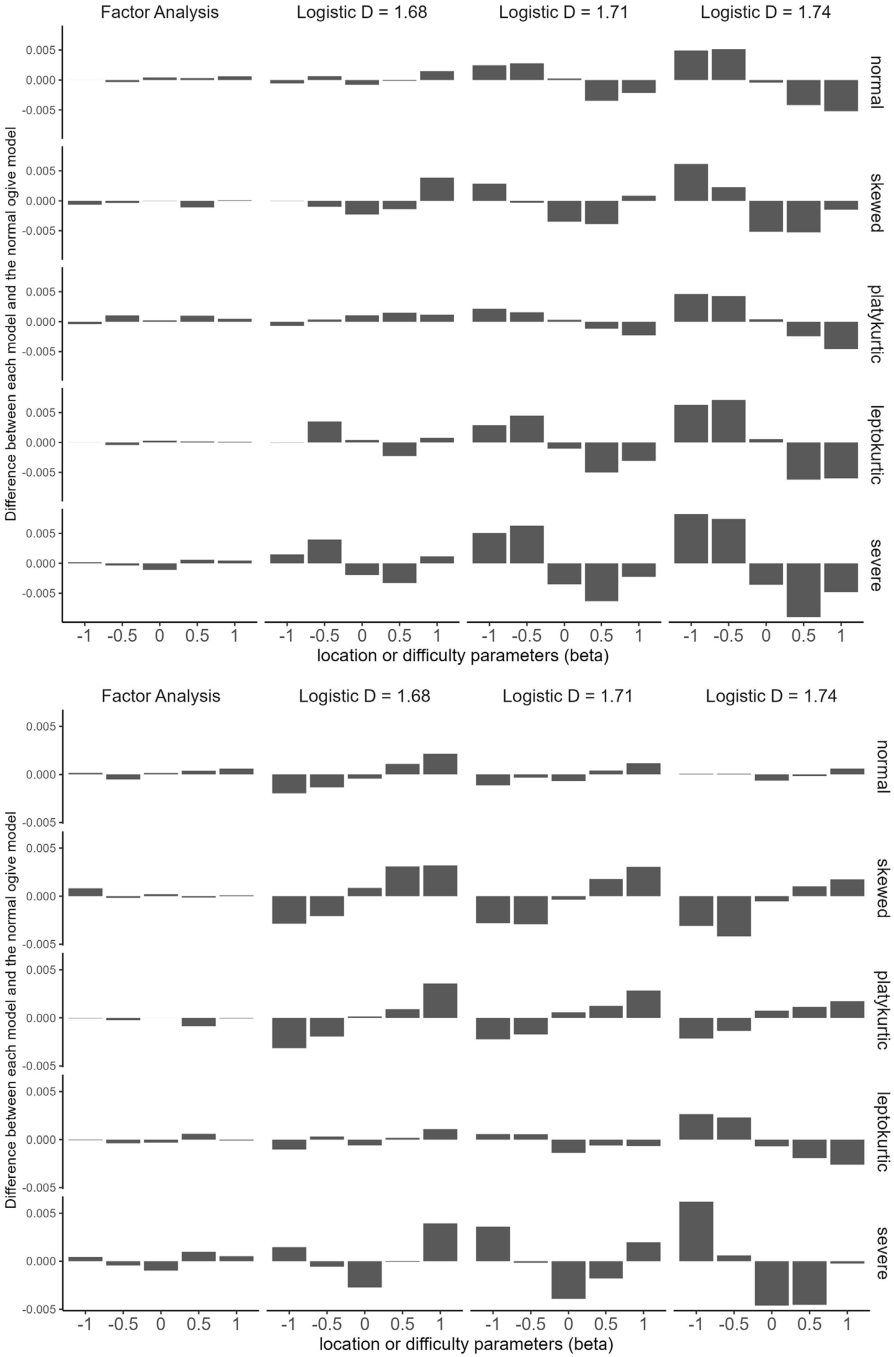
How parameters affect the data generated by each model (Study 4). Top, discrimination parameter (alpha) =0.6; bottom, discrimination parameter = 1.4.

Figure 4 summarizes the results. The FA model produces data that are almost indistinguishable from those of the normal ogive model across all distributions and parameter values. However, logistic models produce distinguishable data according to scaling constants, distributions, and parameter values.

#### Explanation of unexpected results

##### Differences between the FA and logistic models

The way the FA model approximates the normal ogive model differs from the way the logistic model approximates the normal ogive model. The FA model generates data approximate to those of the normal ogive model for any combination of parameters. The logistic model exploits the symmetry of the distribution. For example, consider the model with *D* = 1.71 under the normal condition at the top of Figure 4. If the location parameters are −1 and − 0.5, this model generates more responses in category 1 than does the normal ogive model. However, if the location parameters are 0.5 and 1, this model generates fewer responses in category 1 than does the normal ogive model by approximately the same amount. That is, the logistic model approximates the normal ogive model by making the excess on one side offset the shortage on the other side. However, the excess does not exactly match the shortage. For example, if we look at the model with *D* = 1.68 under the platykurtic condition at the top of Figure 4, there are four excesses and one shortage.

##### Skewness and polytomous data

The excess-offsets-shortage approach works well if the following conditions are met: (1) the distribution has zero skewness (i.e., symmetric) and (2) the data are dichotomous. The nonzero skewness of a distribution causes an excess and shortage mismatch. Figure 4 shows that a skewed distribution makes the data difference between the logistic and normal ogive models asymmetric and irregular. Study 4 also provides clues as to why the blind test results of polytomous data in Study 3 were worse than those of dichotomous data. If there are only two response categories, excess on one side offsets shortage on the other, but if there are multiple response types, it is difficult for this mechanism to work.

##### Effects of parameters

Figure 4 suggests that the location and discrimination parameters can affect the optimal *D*. There can be main effects as well as interactions between them.

## Overall discussion

### Summary of the results

This study examined the interchangeability between the FA model and two IRT models (i.e., logistic and normal ogive). First, the equivalence of the FA and normal ogive models proved under the assumption of normality (Takane and de Leeuw, 1987) is extremely robust, even to severe normality violations. This study compared the results of the two models from various angles but could not find a difference beyond the level attributable to sampling error. Second, the interchangeability of the logistic and normal ogive models is complex and depends on several variables. No single scaling constant maximizes the interchangeability of the two models. The best-performing scaling constant depends on the purpose of using the model (e.g., parameter estimation or data generation), the number of response categories (i.e., dichotomous or polytomous), and the distribution of latent variables (e.g., normal or severely nonnormal).

### Interchangeability between FA and normal ogive models

#### Exploiting the transformation formula

The results suggest that we should exploit Takane and de Leeuw’s (1987) transformation formulas more aggressively than we currently do. If we have two models that give us the same result, we can cherry-pick them as needed. For example, one (e.g., FA) may be faster to compute than the other (e.g., normal ogive), or it may be easier to obtain the necessary software. For example, for parameter estimation, the FA model is likely to be much faster than the normal ogive model due to the functional complexity of the normal distribution. In this case, we can use the easier or faster model to obtain the result and then transform it to the other’s result. The interchangeability between the two models can also be used to improve pedagogical effectiveness. For example, some students are more familiar with the FA model than the IRT model, and others are not. Illustrating an unfamiliar model with a familiar one helps students learn a new model easily. We have underused Takane and de Leeuw’s formulas relative to their potential.

#### Renaissance of the normal ogive model

The first IRTs used the normal ogive function (Thurstone, 1928; Lord, 1952; van der Linden, 2016). The late-discovered logistic model has become more popular than the established normal ogive model because the former is much easier and faster to compute than the latter. For example, two decades ago, computing the normal ogive model was “time-consuming” (Baker and Kim, 2004, p. 16) even on a modern computer of the time, whereas the logistic model could be computed on a pocket calculator. Similar differences are still reported today. For example, a comparison of commonly used R packages showed that estimating the normal ogive model by using the MCMCirtKD package was 19 to 512 times more time consuming than estimating the logistic model using the mirt package (Chalmers, 2012). However, the results suggest that the normal ogive model is no longer more time-consuming than the logistic model. As described earlier, after obtaining the results from the FA model, we can use Takane and de Leeuw’s formulas (Equation 1) to transform them to those of the normal ogive model, which is likely to take a similar amount of time as estimating the logistic model. The irt.fa and fa2irt functions in the psych package (Revelle, 2022) can be used to facilitate this transformation.

Even without this transformation, some software computes the normal ogive model in a fraction of the time, especially for Bayesian IRT (Albert, 1992; Fox, 2010). Furthermore, advances in hardware and software will further narrow the computation time between the two models to a negligible level. As the mutable issue diminishes, an immutability issue arises. The results suggest an advantage of the normal ogive model over the logistic model; the former is fully interchangeable with the FA model, while the latter is not. Therefore, it is easier for the FA and normal ogive models to constitute a generalized model than it is for the FA and logistic models. We also need to be consistent. Statistical models conventionally assume a normal distribution, so describing item responses by using a normal distribution is consistent with this convention (Savalei, 2006). The results suggest that the normal ogive model should be more highly regarded and used more often than it currently is.

Notably, this study did not examine the performance of the three models, so it is inappropriate to conclude that one is better than the other. The results have little to say about which model should be chosen by users who use the models in isolation. However, the results do suggest that users who value interchangeability between models have reason to prefer FA and normal ogive IRT models over logistic IRT models.

### Interchangeability between the normal ogive and logistic models

#### Repositioning the scaling constant

##### From constant to variable

A constant is fixed and unchanging. For example, π is 3.1416 anytime and anywhere. Scholars have used the constant *D* = 1.702 in this sense. Just as no one challenges π  = 3.1416, few scholars have proposed that *D* should be a different value. An exception is Savalei (2006), who argued that the scaling constant she obtained, 1.749, should replace the existing constant, not that these constants should be chosen on a case-by-case basis. In other words, existing studies have assumed that one constant fits all. The results present a new perspective. The optimal scaling constant depends on the purpose of using the model and the number of response categories, and users should choose a suitable scaling constant based on these variables. The scaling ‘constant’ is a variable, not a constant.

##### Omitting *D* from the model

Redefining the nature of the scaling constant raises the need to reconsider its role in the logistic model. A model exists to explain the most with the least, so it should not include an element that does not contribute to explanatory power. From this perspective, the scaling constant *D* is unnecessary in a logistic model because including or omitting *D* does not affect the model’s explanatory power. Today, some scholars omit *D* from their logistic models, and others include it. Even the latter scholars seem to do so reluctantly to maintain historical consistency. For example, Wirth and Edwards (2007) argued that “[i]t is important to note that *D* is something of a historical artifact” (p. 61). Baker and Kim (2004) also stated that “[t]he use of the 1.702 multiplier is a carry over from an earlier time when the normal ogive was the standard” (p. 16). In any case, the mixed use of the two styles creates inconvenience and confusion for users, e.g., by causing users to make mistakes when comparing studies. We should have a clear answer as to whether it is desirable to include *D* in the logistic model and unify the two styles accordingly.

The results answer this question by showing that *D* is not only unnecessary but also changing. Let us suppose some textbook authors choose to describe the logistic model in the traditional Birnbaum style of including *D*. Perhaps their plan is to simply state that *D* is 1.7 or 1.702, which takes up very little space. However, textbooks that maintain this style may be criticized for holding an outdated view whenever new research suggests that *D* should be something other than 1.702. That said, a lengthy discussion of *D* in the first introduction to a model can be disruptive to the flow. An easy solution to this dilemma is to omit *D* from the model.

##### Segmenting users

Even if we omit *D* from the logistic model, we should not ignore the original intent of its inclusion. Whereas users who use the logistic model alone may be uninterested in its interchangeability with other models (i.e., FA or normal ogive), others (e.g., meta-analysts) may be interested in accurately comparing the results obtained from different models. This study argues for segmenting users based on these different needs. Most users find it convenient to use a model with *D* omitted. Those who do care about interchangeability should care about it in a more sophisticated way than today’s approach of relying on “a historical artifact” (Wirth and Edwards, 2007, p. 61). Therefore, we need to know what factors influence the optimal scaling constant, regardless of whether we include it in the model itself or perform a separate transformation.

#### Factors affecting interchangeability

As is often the case with distributions with different kurtosis (Dyson, 1943), the logistic function deviates four times from the normal ogive function (i.e., at X≈ –2, −0.5, 0.5, and 2). Since these two functions are bilaterally symmetric, we can simplify the four errors into two types, the tails (i.e., X≈ –2 and 2) and nontails (i.e., X≈ –0.5 and 0.5). No scaling constant can decrease both errors; decreasing one increases the other. The best approach is to weight the two in proportion to their importance. However, the fact that their relative importance depends on several conditions complicates matters.

The conditions that increase the relative importance of the tail errors compared to the nontail errors are (1) if the model is used for parameter estimation and (2) if the data are polytomous. The condition that decreases the relative importance of the tail errors is (3) if the model is used for data generation and (4) if the data are dichotomous. The more important the tail errors are, the larger the scaling constant that should be used, and vice versa.

The conditions increasing the interchangeability between the logistic and normal ogive models are (A) dichotomous data, (B) zero skewness of the latent variables, and (C) nonpositive kurtosis of the latent variables. The conditions decreasing the interchangeability between the two models are (D) polytomous data, (E) nonzero skewness of the latent variables, and (F) positive kurtosis of the latent variables.

##### Purpose of using a model

Users typically implement a model for parameter estimation. Parameter estimation is sensitive to the tails, and the logistic model assumes heavier tails than the normal ogive model does. Therefore, the logistic model produces parameter estimates with larger absolute values than those of the normal ogive model. Using a scaling constant greater than 1.702 (e.g., 1.739 or 1.753) counterbalances the logistic model’s tendency to overinterpret the tails.

If a model is used to generate data, the optimal scaling constant depends on what the data are to be used for. First, if users plan to use the data for parameter estimation, they should choose a scaling constant greater than 1.702 (e.g., 1.739 or 1.753), just as they did for parameter estimation. Using these scaling constants undergenerates extreme data, which offsets the tendency to overinterpret extreme data in parameter estimation. Second, if users generate data for purposes other than parameter estimation, they should use smaller scaling constants than those just mentioned. The goal is not to undergenerate extreme data but to generate data approximate to those of a normal ogive model.

##### Dichotomous or polytomous data

Birnbaum (1968) originally proposed the scaling constant for dichotomous data. Subsequent scholars have used the same scaling constant (De Ayala, 1994) when proposing logistic models for polytomous data (i.e., graded response models), but few scholars have suggested that a different scaling constant should be used. The results suggest that the optimal scaling constant for dichotomous data is not optimal for polytomous data. A larger scaling constant should be used for polytomous data than for dichotomous data. The difference between the optimal scaling constants of the two data types differs from study to study: a difference of 0.014 in Study 2 and a difference of 0.02 in Study 3 (in the case of a normal distribution).

The importance of the tails differs between dichotomous and polytomous data. In dichotomous data, every response category has its own tail. For example, response category 0 has a left tail, and response category 1 has a right tail. An overgeneration of response category 0 on the left tail can fully cancel out an undergeneration of that response category on the right tail. Therefore, in dichotomous data, nontail errors are relatively more important than tail errors. Moreover, having overgeneration offset undergeneration helps logistic models to be interchangeable with normal ogive models.

However, response categories in polytomous data specialize in the tails or nontails. For example, response categories 0 and 4 have tails, but response categories 1, 2, and 3 do not. An overgeneration of the response category 0 on the left tail cannot fully cancel out an undergeneration of that response category on the right tail. Therefore, there is a strong need to reduce errors in the tails to which response categories 0 and 4 belong. Moreover, since it is difficult for overgeneration to offset undergeneration, the logistic model is less interchangeable with the normal ogive model in polytomous data than it is in dichotomous data.

##### Distribution of latent variables

Latent variables are often assumed to follow a normal distribution. Few scholars have suggested that a violation of the normality assumption can affect the interchangeability between the logistic and normal ogive models or the optimal scaling constant. This study examined the effect of nonnormality by dividing it into skewness and kurtosis. First, a normality violation decreases the interchangeability between the logistic and normal ogive models (Study 3). An exception is the platykurtic distribution, in which case the results are mixed. Among normality violations, violations of skewness have a greater effect than violations of kurtosis. The logistic and normal distributions have different kurtosis, but both distributions have zero skewness (i.e., symmetric). Symmetry helps the logistic model produce approximate results to those of the normal ogive model, i.e., a surplus in one tail cancels out a shortage in the other tail. Asymmetry creates a surplus and shortage mismatch.

Second, latent variables that follow a nonnormal distribution require different scaling constants than those that follow a normal distribution. In the case of a platykurtic distribution, scaling constants larger than those of the normal distribution, and in the case of the severely nonnormal distribution, scaling constants smaller than those of the normal distribution produced good results. In the case of skewed and leptokurtic distributions, the results are mixed.

## Concluding remarks

What we see may not be what we get. At first glance, the FA model is different from the two IRT models (i.e., logistic and normal ogive), and the two IRT models are similar. However, the FA and normal ogive models are more interchangeable than expected, whereas the normal ogive and logistic models are not. When a probability distribution is graphed (e.g., Figure 2), the tails are less visible than the nontails, so we are likely to perceive the tails as less important than the nontails. Sometimes the tails are much more important than the nontails, but other times they are not. Larger scaling constants have advantages in the tails, and smaller constants have advantages in the nontails. Therefore, no single constant can provide maximum interchangeability between the logistic and normal ogive models in all situations. Choosing a scaling constant is thus a more complex process than scholars have assumed.

## Data availability statement

The datasets presented in this study can be found in online repositories. The names of the repository/repositories and accession number(s) can be found at: https://github.com/eunscho/FAIRT.

## Author Contributions

EC: Conceptualization, Data curation, Formal analysis, Funding acquisition, Investigation, Methodology, Visualization, Writing – original draft, Writing – review & editing.

## Funding

The author(s) declare financial support was received for the research, authorship, and/or publication of this article. This work was supported by the Ministry of Education of the Republic of Korea and the National Research Foundation of Korea (NRF-2021S1A5A2A03061515) and the Research Grant of Kwangwoon University in 2023.

## Conflict of interest

The author declares that the research was conducted in the absence of any commercial or financial relationships that could be construed as a potential conflict of interest.

## Publisher’s note

All claims expressed in this article are solely those of the authors and do not necessarily represent those of their affiliated organizations, or those of the publisher, the editors and the reviewers. Any product that may be evaluated in this article, or claim that may be made by its manufacturer, is not guaranteed or endorsed by the publisher.

## Footnotes

^1^ https://github.com/eunscho/FAIRT
